# Characterization of a Novel 3 Megavolt Linear Accelerator for Dedicated Intracranial Stereotactic Radiosurgery

**DOI:** 10.7759/cureus.4275

**Published:** 2019-03-19

**Authors:** Georg A. Weidlich, Mohan Bodduluri, Younes Achkire, Chris Lee, John R. Adler

**Affiliations:** 1 Radiation Oncology, National Medical Physics and Dosimetry Company, Palo Alto, USA; 2 Medical Physics, Zap Surgical Systems, San Carlos, USA; 3 Radiation Oncology, Stanford University Medical Center, Stanford, USA

**Keywords:** stereotactic radiosurgery, self-shielded, intra cranial, 3mv linear accelerator

## Abstract

The purpose of this article is to investigate and characterize from a physics perspective the Zap-X (ZAP Surgical Systems, Inc., San Carlos, CA), a new, dedicated self-contained and self-shielded radiosurgery system, focusing on beam energy and performance, leakage, radiation safety, dose delivery accuracy, regulations, quality assurance, and treatment planning. This investigation is required to establish the mechanical and overall performance specifications of the system and to establish baseline parameters for future clinical usage.

The applied methods include measurements of energy, focal spot size, beam performance, dosimetry, beam data, treatment planning system, leakage radiation, acceptance testing, and commissioning.

The results of the characterization reveal a 3 megavolt (MV) linear accelerator (linac) with a focal spot size of 2 mm, a dose rate of 1,500 MU/min at the isocenter with a dose linearity of 3%, a beam penumbra of less than 3 mm, and beam symmetry of less than 2%. Beam performance, as well as dosimetry characteristics, are suitable for intracranial radiosurgery.

It can be concluded that the system was found to meet safety, accuracy, and performance requirements widely accepted in the radiation oncology and radiosurgery industry. Furthermore, the system was shown to meet the practical, clinical needs of the radiosurgery community.

## Introduction

The Zap-X is a new, dedicated self-contained and self-shielded radiosurgery system developed and manufactured by ZAP Surgical Systems, Inc. of San Carlos, California. This device is intended for stereotactic radiosurgery (SRS) treatment of benign and malignant intracranial and cervical spine lesions. A 3 megavolt (MV) S-band linear accelerator (linac) is the source of therapeutic radiation. Akin to a large gyroscope, the linac is mounted within a combination of yoked gimbals with attached radiation shielding, each of which accurately rotates around a common isocenter. This mechanical construct enables the linac beam to potentially crossfire from approximately 2π steradians of solid angle, as is ideally required for cranial SRS.

Accurate therapeutic beam positioning is accomplished through the dual-axes, independent rotations of the accelerator and precise movements of a robotic patient table. Most components needed to produce the beam, such as the radiofrequency power source, waveguiding system, and beam triggering electronics, as well as significant radiation shielding, are mounted on or integrated into the rotating spherical patient treatment chamber. The patient is supported on a moveable treatment table that extends outside the iron sphere but which itself is also enclosed by additional radiation shielding during radiosurgery. This table shielding consists of a rotary iron shell and pneumatic door on a steel frame.

## Technical report

**Figure 1:**
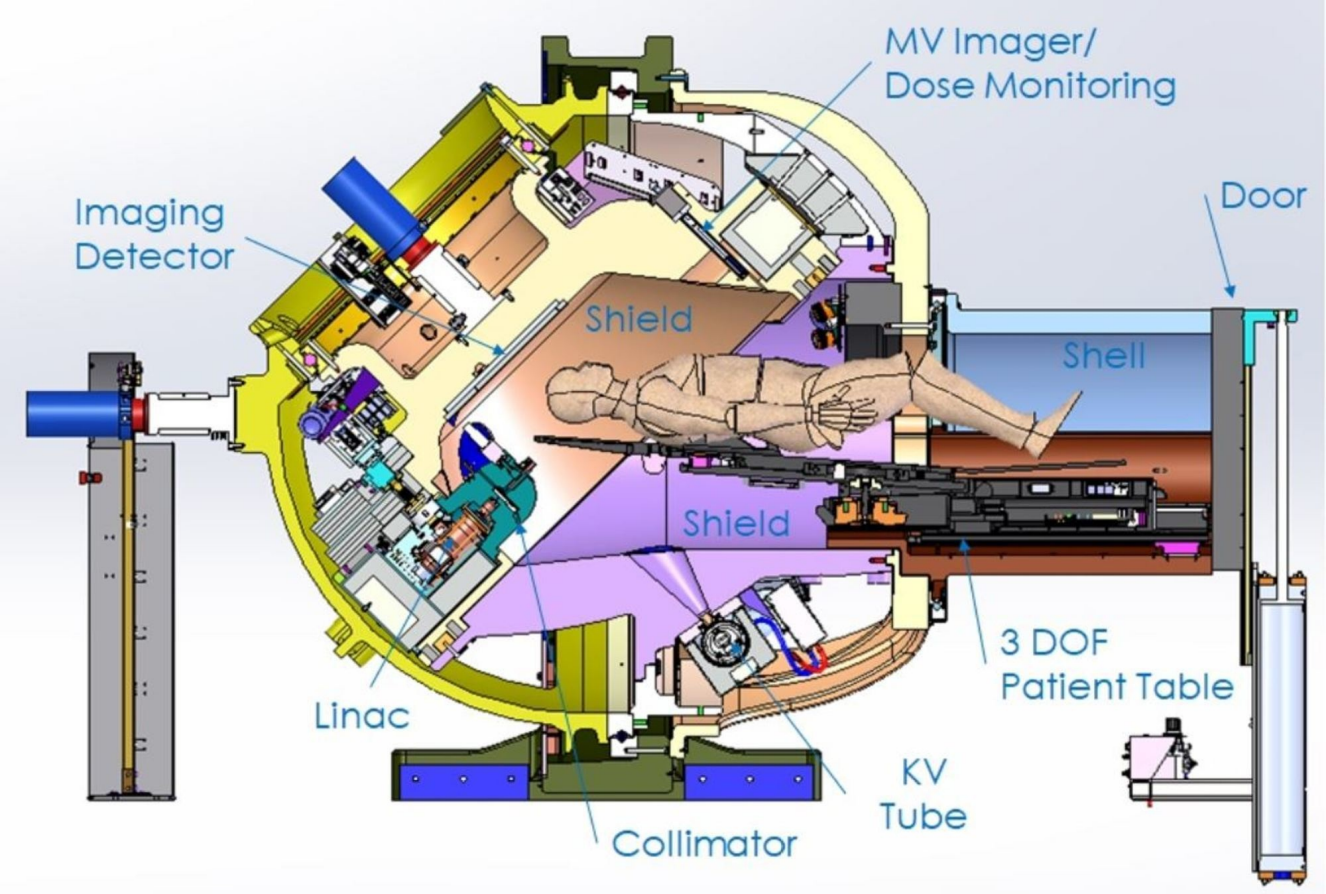
Cross-sectional view of Zap-X MV: megavoltage: DOF: degree of freedom; KV: kilovoltage

**Figure 2:**
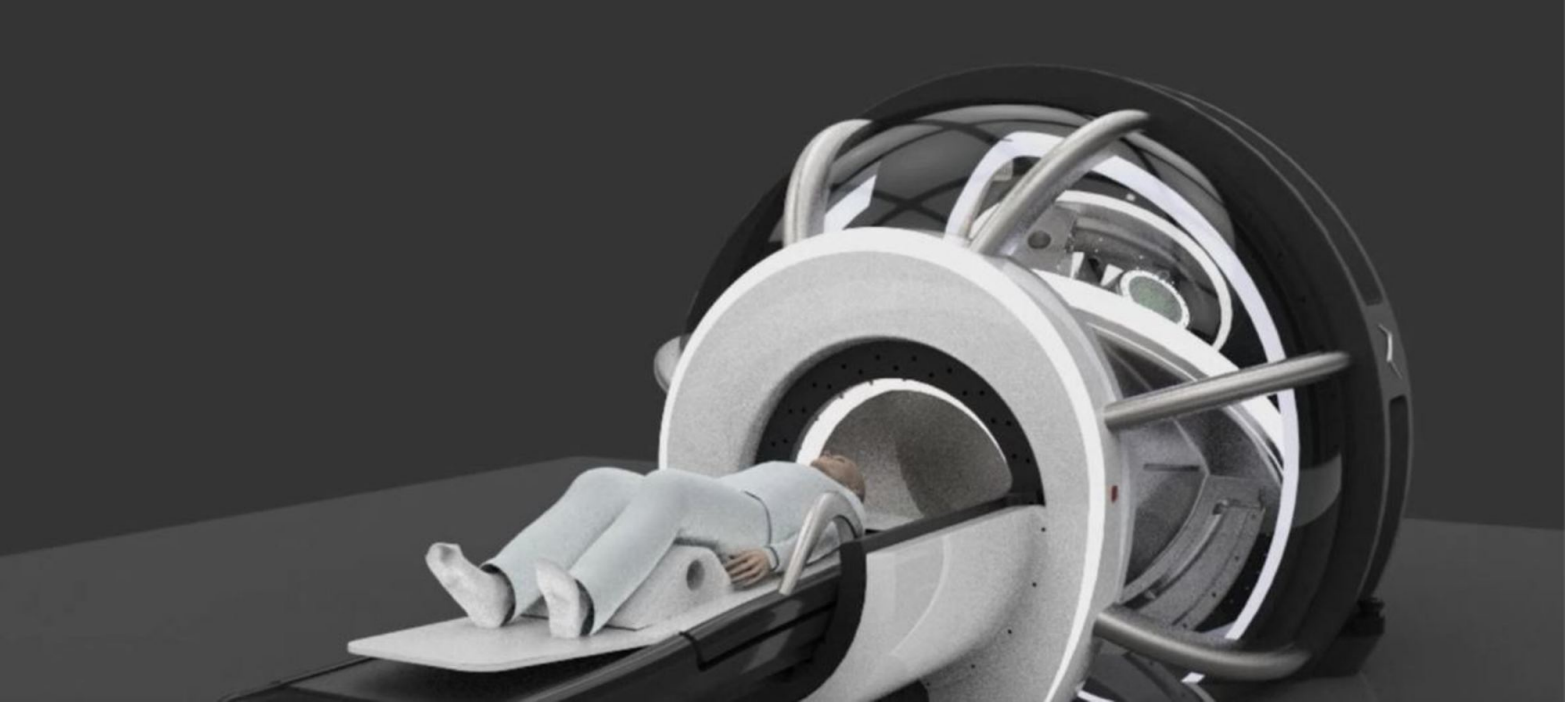
Room's eye view of Zap-X with patient in the loaded position

**Figure 3:**
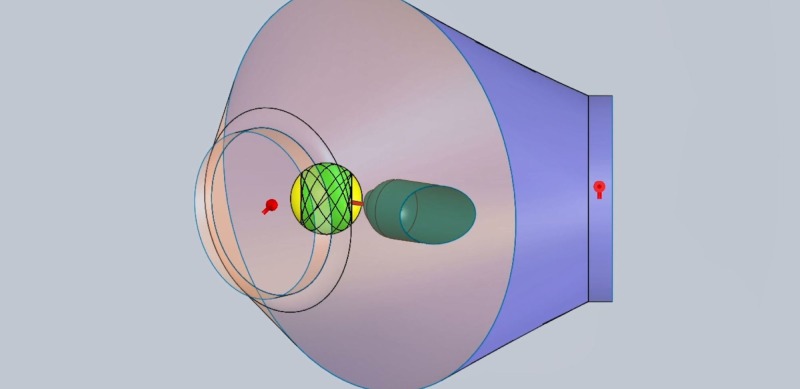
Solid angle shaded in green represents beam coverage

The Zap-X is shown in Figure 1 and has been described in more detail in previous publications [1-3]. Figures 2-3 below show marked up views of the Zap-X system in operation, as well as the beam coverage.

To characterize the Zap-X system, the following subjects were investigated and/or implemented:

1) System shielding

2) The energy of the linear accelerator, thereby enabling both the nominal energy and accelerating potential to be derived

3) The focal spot size, measured with a custom-designed focal spot size 'camera', that allowed exclusively primary radiation to pass through a group of tightly stacked tungsten leaves

4) The linac collimator leakage radiation

5) A comprehensive radiation survey outside the shielded sphere and housing of the Zap-X with derivation of the expected annual dose

6) The reproducibility and linearity of the dose monitor ionization chamber

7) The Zap-X system compliance with International Electrotechnical Commission (IEC) regulations [4]

8) Beam Performance Specifications and Acceptance Testing

9) A complete set of beam data as required for the Treatment Planning System

10) A comprehensive Quality Assurance (QA) Program

Each individual task was addressed to establish the most descriptive representation of the linear accelerator which will ultimately help the practicing user to better understand the capabilities, limitations, and characteristics of the Zap-X system.

System shielding design

The Zap-X system consists of two shields that move independently of each other on axes that are separated by an angle of 45 degrees. The design method is applied to determine the thickness of the Zap-X shield to which the linac/collimator is attached. Once the thickness profile has been calculated, the largest thickness profile is applied to each cross-section of the shield.

Figure 1 above illustrates the components that constitute the radiation shielding of the Zap-X system, including the collimator assembly, the beam stop aligned with the treatment beam/collimator beam axis, a moving shield which constitutes the secondary shielding to protect the facility employees and the general public, the patient inside the self-shielding system, and the boundary that limits the controlled area.

In addition to aiming the radiation treatment beam onto the patient’s head or neck target, the system shall provide the proper radiation shielding to limit the radiation exposure to members of the public and facility employees to an acceptable level. The Zap-X self-shielding is designed to achieve a control area limit at a radius of no more than 3 meters from the isocenter. The system shall also limit the leakage of radiation to the patient to less than 0.1%. The multi-collimator revolver assembly also serves to shield against this leakage radiation. To achieve such radiation exposure limits, the moving and static shields shall be designed based upon the results from a Monte Carlo simulation model. The input to this model shall be the linac energy (MV) and the maximum dose rate (cGy/min). The maximum dose for the general public (i.e., belonging to the uncontrolled area) required by the National Council on Radiation Protection and Measurement (NCRP) is 1 millisievert (mSv)/year, which is equivalent to 100 milliroentgens (mR)/year. For a total of 2,000 work hours per year, this will result in 0.05 mR exposure in any one hour.

The Monte Carlo simulations predicted the angular distribution for the first, second, and third tenth-value layers for the radiation scattered from the target of the linac (shielded by the collimator assembly) and the angular distribution for the first, second, and third tenth-value layers for the radiation scattered from the patient head. For the medical device shielding design requirement, one should assume the worst-case conditions and define the maximum dose rate (D), the number of patients (Np) per week, the average treatment time (T min/patient), the qualification and calibration time to not exceed a certain duration (Tqc) per week, the total workload (W = Np x T + Tqc hours per week), the utilization (U), add a safety factor (SF), and number of weeks (Nw) of operation in one year.

The equivalent radiation on time per year is calculated by applying the following formula: U x W x SF x Nw (h/year). From this result, the admissible radiation exposure limit at the controlled area boundary can be determined. This is the main requirement used to design the shielding thickness of the device and determine the radius of the spherical control area.

With the first angular distribution (1), the collimator assembly thickness shall be designed/calculated to provide an attenuation of the target scattered radiation of the order of 0.001%. To increase the treatment beam coverage around the patient’s head, the collimator assembly was made of tungsten to minimize this required thickness. The thickness of the moving and static shield around the patient was determined based on the leakage radiation from the collimator and the scattered radiation from the patient.

Shielding materials used were high-density materials, such as tungsten, lead, and iron. Iron is the main material used for the secondary scattering shields. Lead and tungsten were used whenever the designed ductile iron thickness exceeded the limits of the mechanical subsystem space work. To evaluate the required shielding thickness profile, a polar coordinate system centered on the isocenter is used and the general public radiation limit (1 mSv) boundary is discretized as a function of the angle. For every point on that boundary, one can solve for both the thickness to attenuate the collimator radiation leakage and for the thickness to attenuate the scattered radiation from the patient. Since the system is symmetric around the beam axis, the thickness can be evaluated for an angle range between 0 degrees aligned with the direction of the radiation beam and 180 degrees. Hence, the thickness profile for the first shield (i.e., supporting the linac/collimator) is revolved around the radiation treatment beam axis to obtain the three-dimensional (3D) shielding volume/chamber.

Once the thickness profile has been calculated, the largest thickness profile for the worst-case radiation scenario is applied to each cross-section of the second shield that moves relative to the first one. The Zap-X System shields were designed using those calculated shielding profiles and a first production pilot system was built.

Linear accelerator energy characterization 

**Figure 4:**
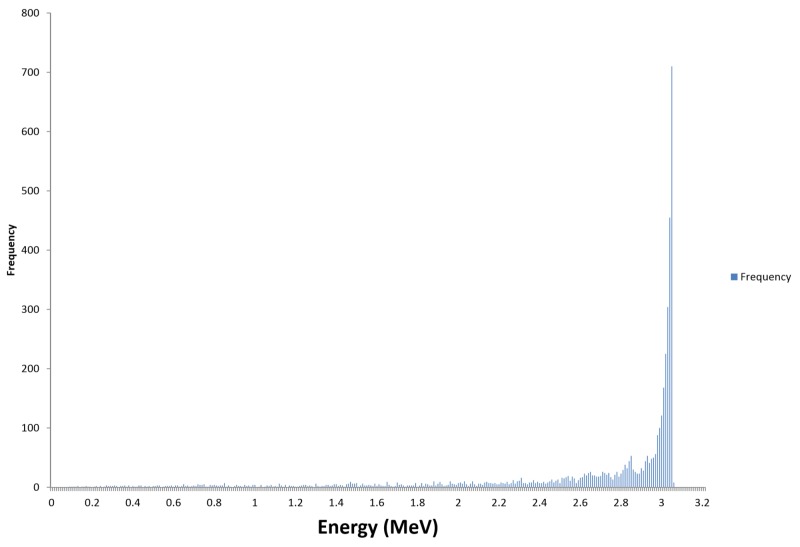
Calculated 3 mega electron volt electron energy spectrum incident on x-ray target using Monte Carlo simulation

**Figure 5:**
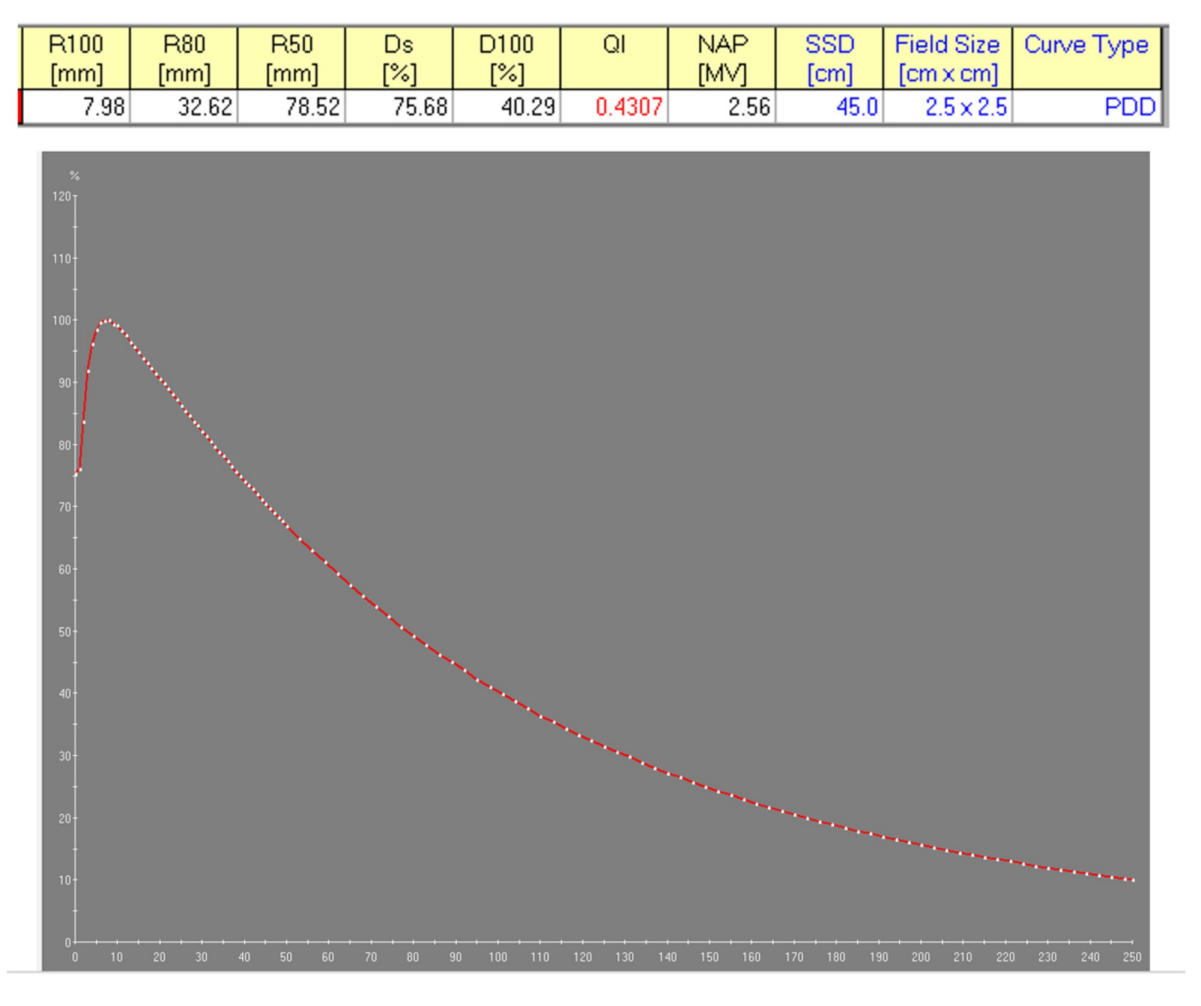
Measured percent depth dose of 40.29% for the Zap-X 3.0 MV photon beam R: reading; Ds: surface dose; D100: dose at 100 mm depth; QI: quality index; NAP (MV): nominal accelerating potential (megavoltage); SSD: source surface distance

The linear accelerator is a key component of the Zap-X System as it produces the therapeutic photon radiation used for patient treatment. Its penetrative quality was carefully matched to be best suited for the treatment of intracranial targets. To obtain the lowest whole brain dose (and yet maintain sufficient beam penetration for therapeutic linear accelerators), it is desirable to obtain a percent depth dose (PDD) on the central axis (cax) of 60% to 70% at the most commonly used treatment depth. As the common depth for intracranial radiosurgery is approximately 5 cm, the 3.0 MV photon beam with a 66% PDD at this depth appears well-matched to the treatment of intracranial SRS targets. This photon radiation is produced by the bremsstrahlung interaction of the accelerated electrons with the nuclei of the tungsten material of the target. The Linac beam was measured using a depth dose scan in a PTW 3D water phantom (Physikalisch-Technische Werkstaetten, Freiburg, Germany) at 100 cm source-to-surface distance (SSD) and reference data from the British Journal of Radiology, Supplement 17 [5]. The electron energy spectrum and the resulting photon bremsstrahlung PDD are shown in Figures 4-5, respectively.

Focal spot size measurement and beam penumbra

A focal spot size camera was designed to determine the size of the photon beam focus. The focal spot size has a direct impact on the geometric penumbra of the beam and a small penumbra will allow the creation of steep dose gradients at the target to critical anatomical structure interface. A focal spot size significantly less than 2 mm could cause structural damage to the target due to the inability for sufficient heat dissipation in the target material and temperatures that exceed the melting point.

**Figure 6:**
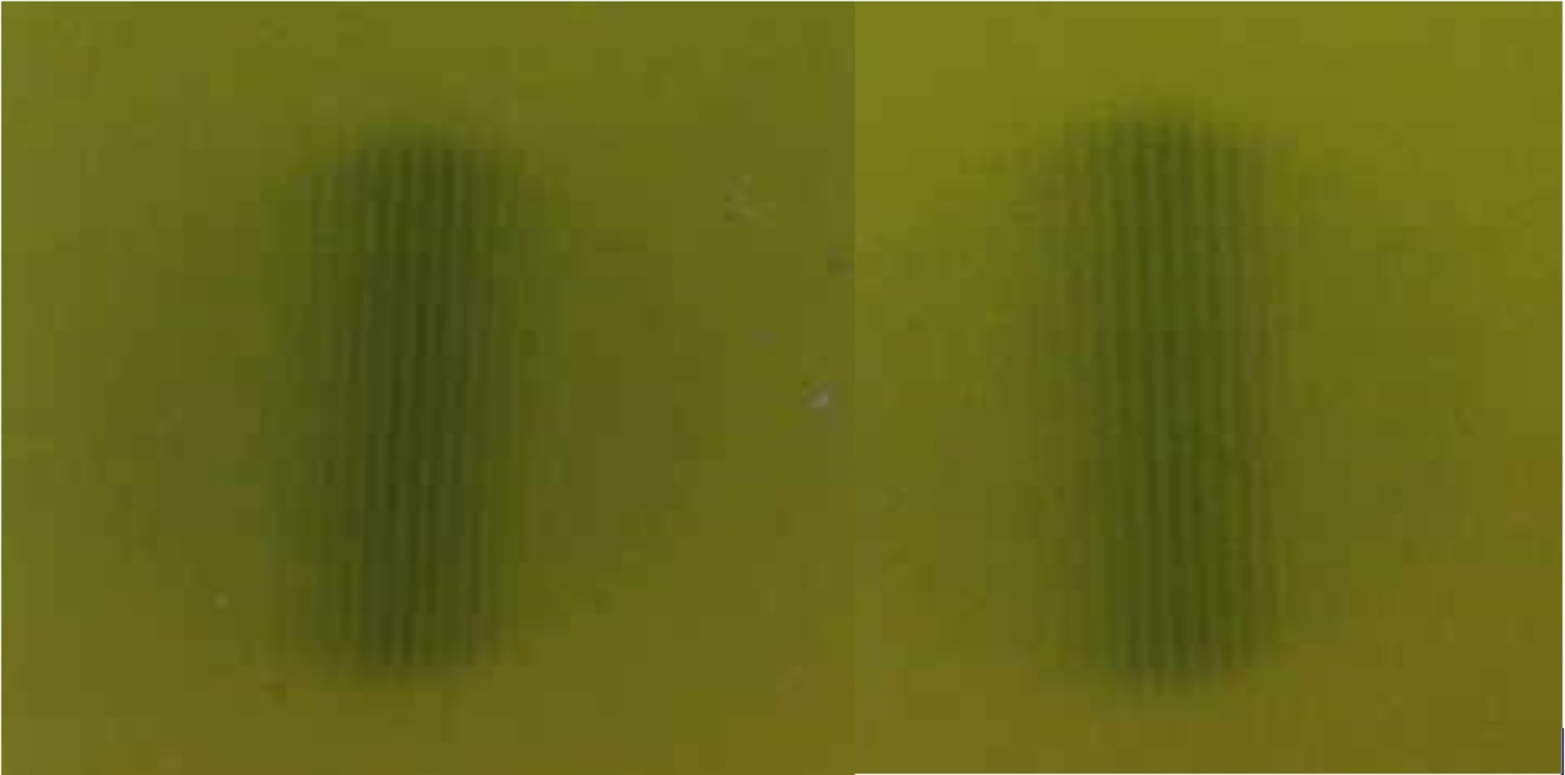
Focal spot size exposures in the in-plane and cross-plane orientations

**Figure 7:**
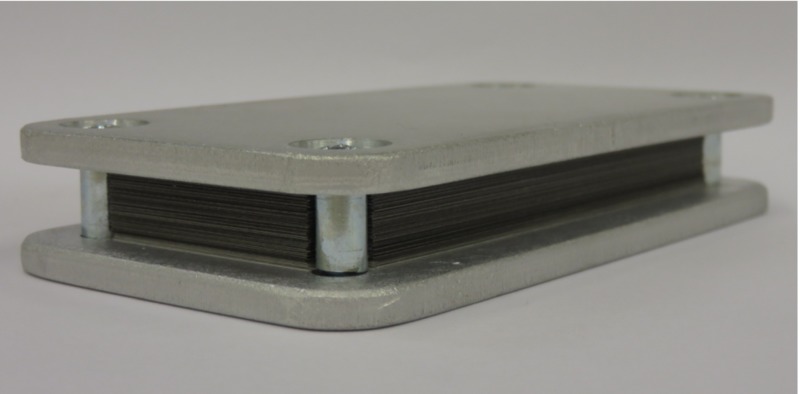
Focal spot size camera

The camera consisted of 100 leaves of 0.1 mm thick tungsten (W) sheets, alternating with 0.1 mm paper spacers, both having a size of approximately 5 cm x 8 cm size. The leaves and spacers were tightly held in place by bracketing aluminum plates. The focal spot camera was positioned directly downstream of the collimator wheel of the linac at SSD = 22.8 cm at the upstream edge of the camera. A 7.5 mm collimator was selected, and the focal spot camera was positioned with the long edge of the camera pointing up and the central beam axis along the plane of the leaves. Exposures were made with Gafchromic™ film (Ashland Advanced Materials, Bridgewater NJ) to record the radiographic transmission pattern, both in the plane of the collimator wheel and in the orientation perpendicular to the collimator wheel. The exposures are shown in Figure 6. The focal spot size camera is displayed in Figure 7.

Nine-line pairs were identified which translates to a 1.8 mm focal spot size. As the outer two-line pairs were fading, an uncertainty of +/- 0.2 mm was assigned. The focal spot size can be considered circular.

**Figure 8:**
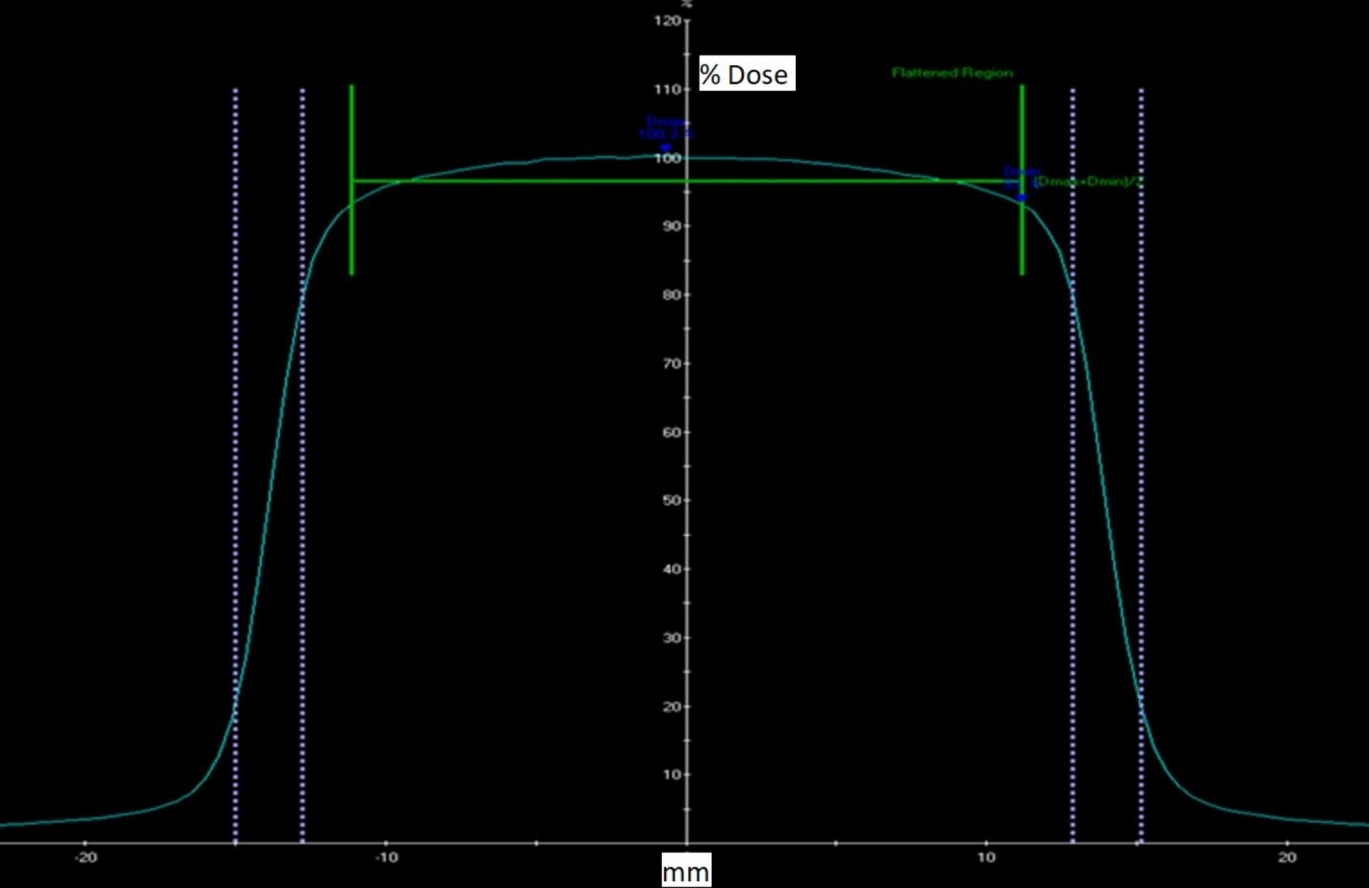
Measured beam profile of 25.0 mm collimator at 5.0 cm depth, with indication of symmetry and penumbra

The beam penumbra was measured for all collimator sizes of 4, 5, 7.5, 10.0, 12.5, 15, 20, and 25 mm at a 5 cm and 10 cm depth at SSD = 45 cm. The beam penumbra was defined as the distance between the 80% to 20% intensity points. Figure 8 below shows the beam profiles for the 25 mm collimator which is the calibration field size.

Table 1 below shows the summary of the penumbra values for all collimator sizes at 5.0 cm and 10 cm depth.

**Table 1:** Penumbra values in mm for all collimator sizes at 5.0 cm and 10.0 cm depth The higher value of the wheel plan and orthoplane penumbra are recorded. Wheel plane is defined as the plane that contains the collimator wheel. The orthoplane is perpendicular to it.

Collimator size	4 mm	5 mm	7.5 mm	10 mm	12.5 mm	15 mm	20 mm	25 mm
Left Penumbra 5 cm	1.77	1.88	1.94	2.00	2.06	2.11	2.19	2.27
Left Penumbra 10 cm	2.00	2.12	2.21	2.29	2.35	2.43	2.54	2.64
Right Penumbra 5 cm	1.78	1.85	1.93	1.99	2.08	2.11	2.19	2.27
Right Penumbra 10 cm	2.02	2.11	2.19	2.28	2.39	2.44	2.53	2.69

**Figure 9:**
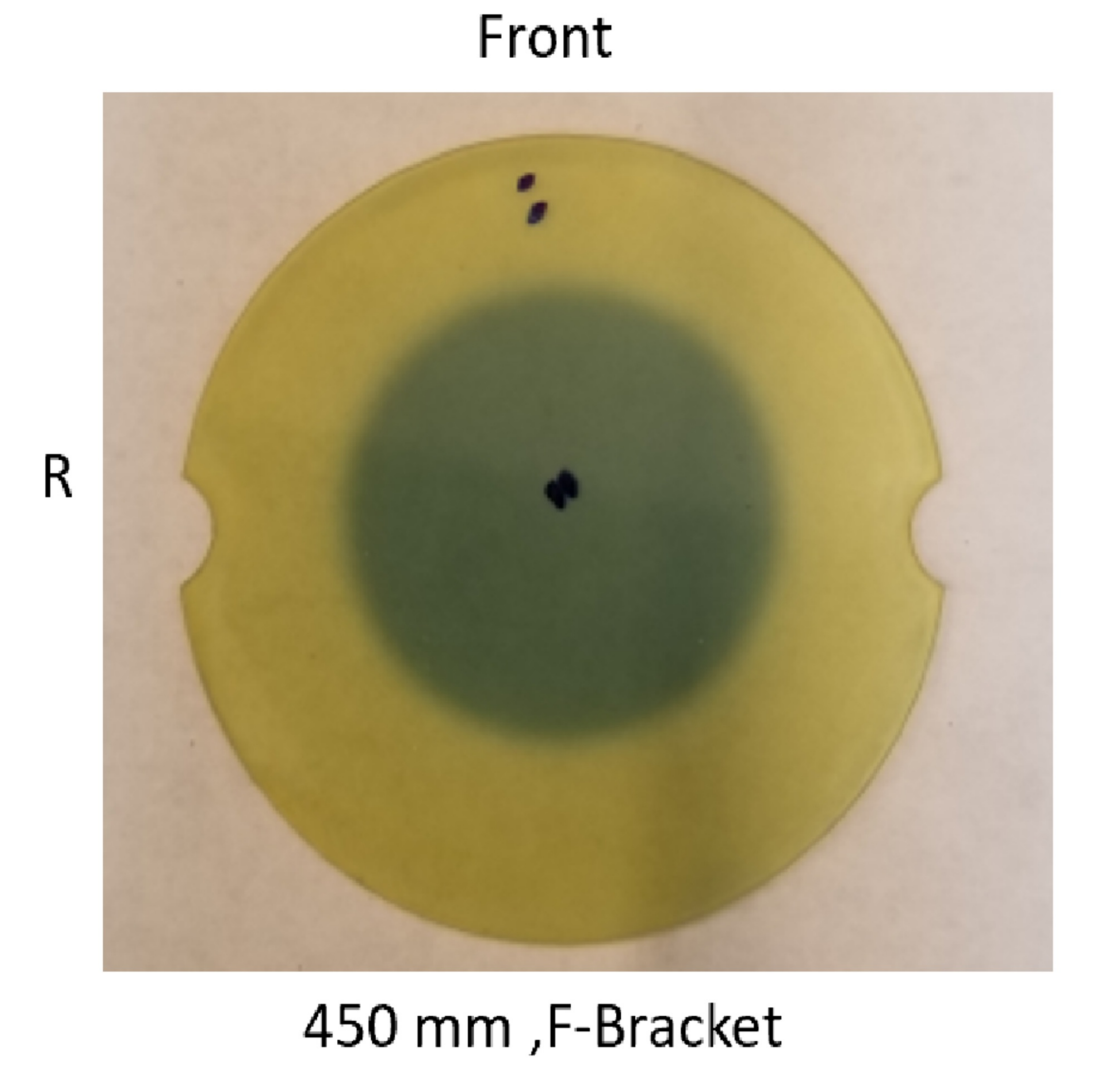
Exposed Gafchromic film for 25 mm collimator field size; dark grey area represents the exposure of the film, prick marks indicate center and orientation of the film

**Figure 10:**
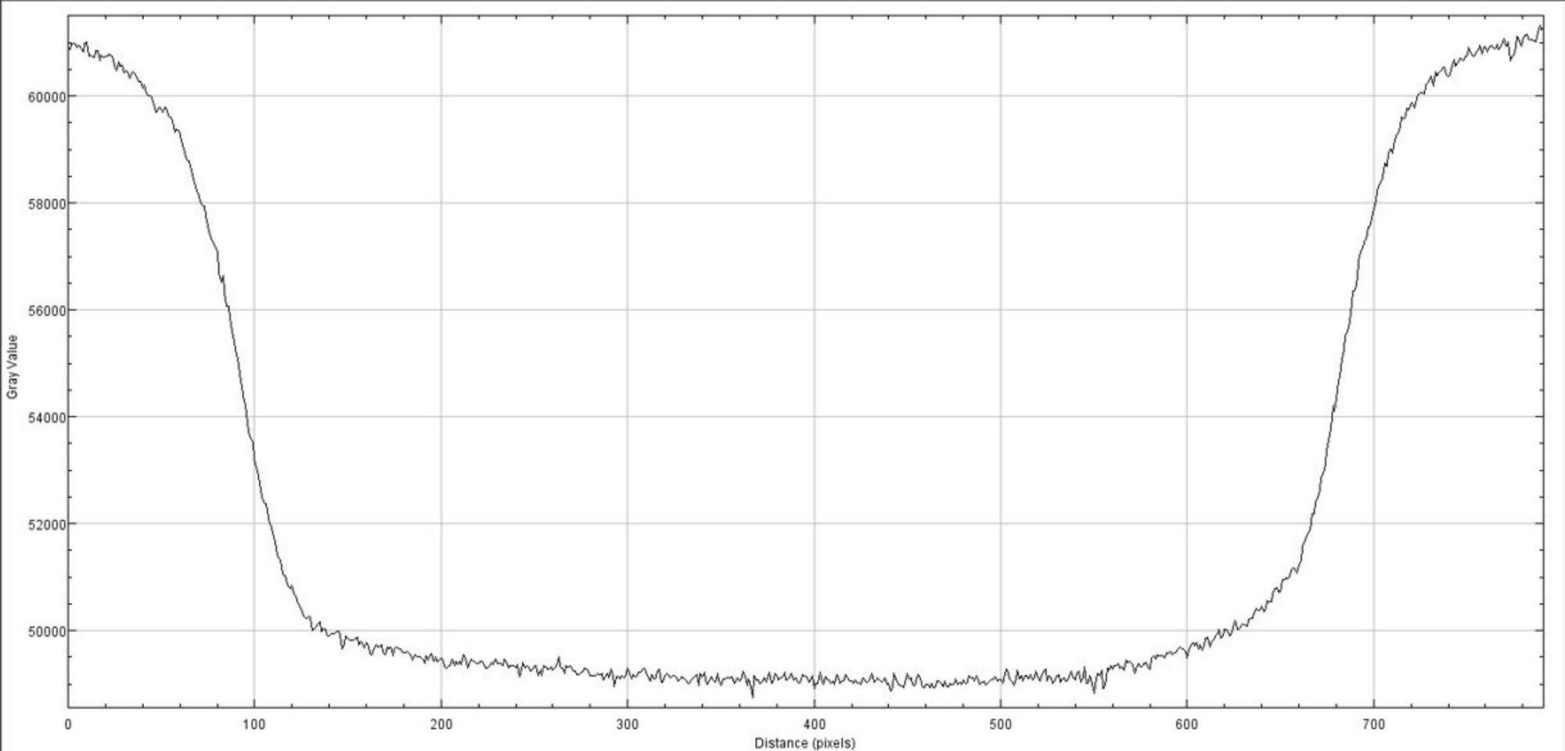
Exposure profile of 25 mm collimator field size on the Gafchromic film

The radiation exposure profile was measured with the Gafchromic film at the isocenter using maximum build-up material (Figure 9). The exposed film was scanned, and the exposed film transmission was plotted in Figure 10.

Linac collimator leakage radiation

As previously described [6], the collimator of the Zap-X system is designed with extremely low leakage values, exceeding the IEC 60601-2-1, v. 2014, standards [4]. The leakage radiation was evaluated via measurement at several discrete locations. The measurement equipment consisted of a large volume calibrated leakage chamber from Radcal, Model # 10X6-180, S/N 08-0727 (RadCal Corp., Monrovia, CA) and an ionization chamber-based Victoreen™ survey meter, Model 451 BRYR, S/N 0000003284 (Fluke Biomedical, Cleveland, OH) at various positions in the patient plane and alongside the Zap-X collimator housing. Both chambers were calibrated at an accredited dosimetry calibration laboratory (ADCL). All measurements were performed with a blank collimator selected (meaning the collimator wheel was positioned so that the beam intercepted the solid tungsten material).

**Figure 11:**
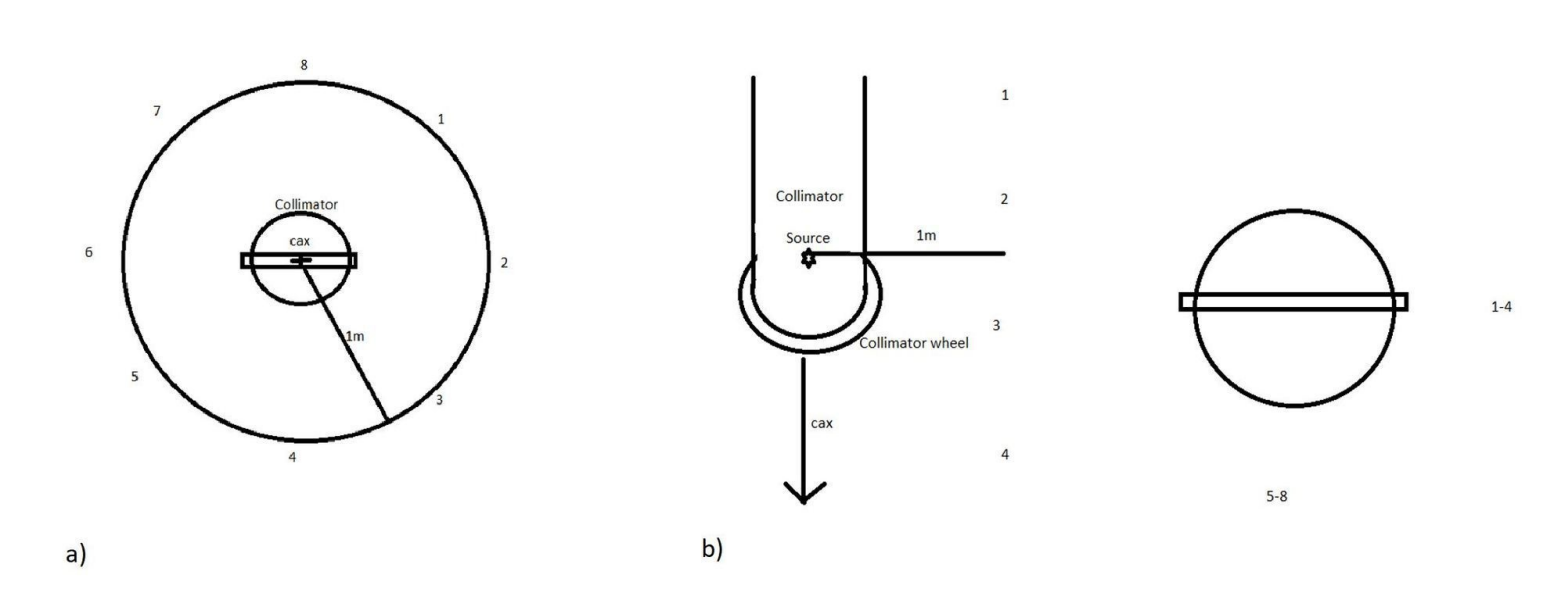
Indication of leakage measurement locations a) in the patient plane and b) along the linear accelerator

In the patient plane, a maximum leakage was measured at station #8 with a value of 10.4 mR. As 1,000 monitor units (MU) were delivered during this measurement, a 1,000 Roentgen (R) exposure occurred on the central axis, and therefore, 10.4 mR corresponds to 0.00104% of the primary beam intensity. A graph indicating the locations is shown in Figure 11a [6]. Along the linear accelerator, a maximum leakage was measured at station #3 with a value of 6.8 mR. As 1,000 MU were delivered during this measurement, a 1,000 R exposure occurred on the central axis, and therefore, 6.8 mR corresponds to 0.00068% of the primary beam intensity. A graph indicating the locations is shown in Figure 11b [6].

As the IEC standards require a maximum of 0.1% of leakage radiation in the patient plane and along the linear accelerator, the Zap-X exceeds the requirements of this standard by two orders of magnitude.

Radiation survey and radiation safety

As shown earlier [3], the Zap-X can be considered self-shielded in the sense that under standard heavy operating conditions, at 1 m distance from the perimeter of the Zap-X, the maximum expected radiation equivalent dose level is below 1 mSv/year, which is equivalent to the limit for public exposure.

Standard heavy operating conditions assume that a total of 2,250 patients will be treated for a very busy operation, all with a single fraction. It is assumed that a spherical head (20 cm diameter) will be treated with the target in the center of the sphere at 10 cm depth. For the target, the prescription is 2,500 centiGray (cGy) to 80% isodose, collimator size 25 mm (output factor (OF) 1.0), and depth of 10 cm (tissue-phantom-ratio (TPR) ~0.5). The resulting MUs per treatment is 6,250 MU. With the given patient distribution, a total of 56,250 MU will be delivered per day or 1.406 x 107 MU per year. This translates into 156.25 hrs/year of “Rad On” time; therefore, we will have a utilization factor of 156.25/2,000 = 0.0781. The shielding should be designed for 500 mrem/year or 0.25 mrem/hr. Applying the utilization factor, the allowable instantaneous exposure rate is 3.2 mrem/hr at 1 m. This value is expected to decrease if the utilization of the system will increase. To establish a safe perimeter for radiation workers, when the final design is established, the distance from the ZAP system will need to be established in all directions, at which 3.2 mR/hr is detected.

Only under the most extreme operating conditions (with more than 50 patients being treated per week) would the self-shielding characteristics not hold true. In that case, a reassignment of the area in the immediate vicinity of the Zap-X to occupationally exposed with 5.0 mSv/year would allow the patient census to be quintupled and remain self-shielded for radiation workers.

**Figure 12:**
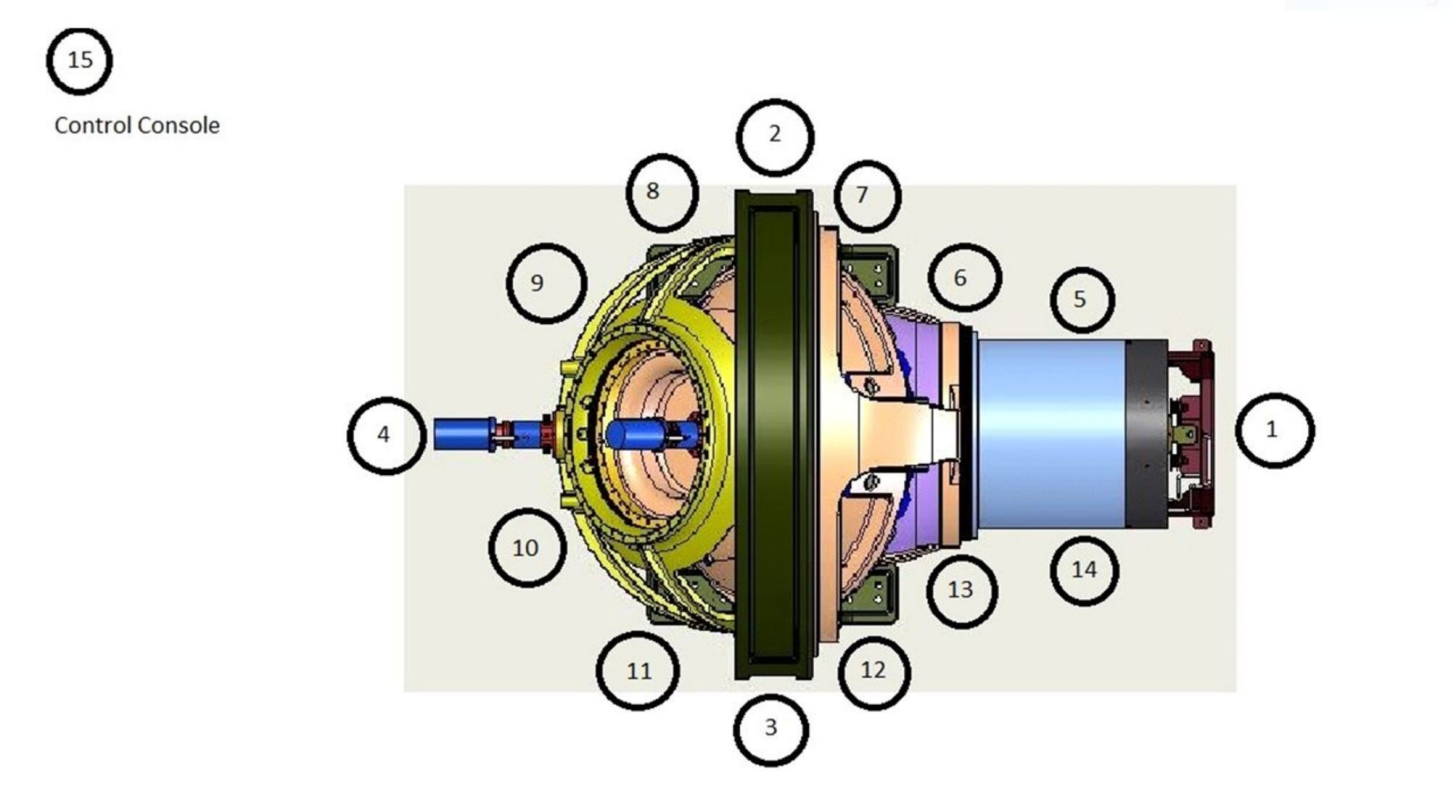
Description of measurement locations for Zap-X radiation survey

The results of the radiation survey are shown in Table 2 [3] as a list of measurement locations and Figure 12 [3] that shows the measurement locations. All projected annual dose values were derived to be below 1.0 mSv which is equivalent to the annual public dose limit.

**Table 2:** Results of radiation survey in units of mR/hr Beam pos: beam position; mR/hr: milliroentgens per hour; U: use factor; D/C: duty cycle; Ann Dose (mSv): annual dose in millisieverts; G: gantry

Location #	Description of Stations	Beam pos.	mR/hr	U	D/C	Ann Dose (mSv)
1	Foot End Table Shield	G = 45	3.0	0.2	0.078	0.936
2	Right Side Main Gantry	G = 45	0.09	0.2	0.078	0.028
3	Left Side Main Gantry	G = 90	0.1	0.2	0.078	0.031
4	Head End of System	G = 45	0.36	0.2	0.078	0.112
5	Table Right	G = 90	0.45	0.2	0.078	0.140
6	Table - Orbit Right	G = 90	0.37	0.2	0.078	0.115
7	Right Gantry	G = 0	0.19	0.2	0.078	0.059
8	Right Gantry	G = 270	1.72	0.2	0.078	0.537
9	Right – Head	G = 45	2.70	0.2	0.078	0.842
10	Left – Head	G = 45	2.80	0.2	0.078	0.874
11	Left Gantry	G = 45	1.70	0.2	0.078	0.530
12	Left Gantry	G = 0	0.57	0.2	0.078	0.178
13	Table - Orbit Left	G = 0	0.84	0.2	0.078	0.262
14	Table Left	G = 0	0.4	0.2	0.078	0.125
15	Control Console	G = 0	0.11	0.2	0.078	0.034

The maximum detected instantaneous exposure rate was detected at measurement station 1 with 3.0 mR/hr. As this value is below the maximum allowable instantaneous exposure rate of 3.2 mR/hr, it is deemed safe for the public.

Dose monitor ionization chamber

The dose monitor ionization chamber measures the amount of radiation delivered to ensure that the dose rate and absolute dose will be delivered as prescribed. Due to the very small maximum field size of 2.5 cm diameter at the isocenter, no flatness or symmetry monitoring is implemented. The dose monitor chamber consists of a dual ionization chamber design with two independently operated units to provide redundancy and safety.

**Figure 13:**
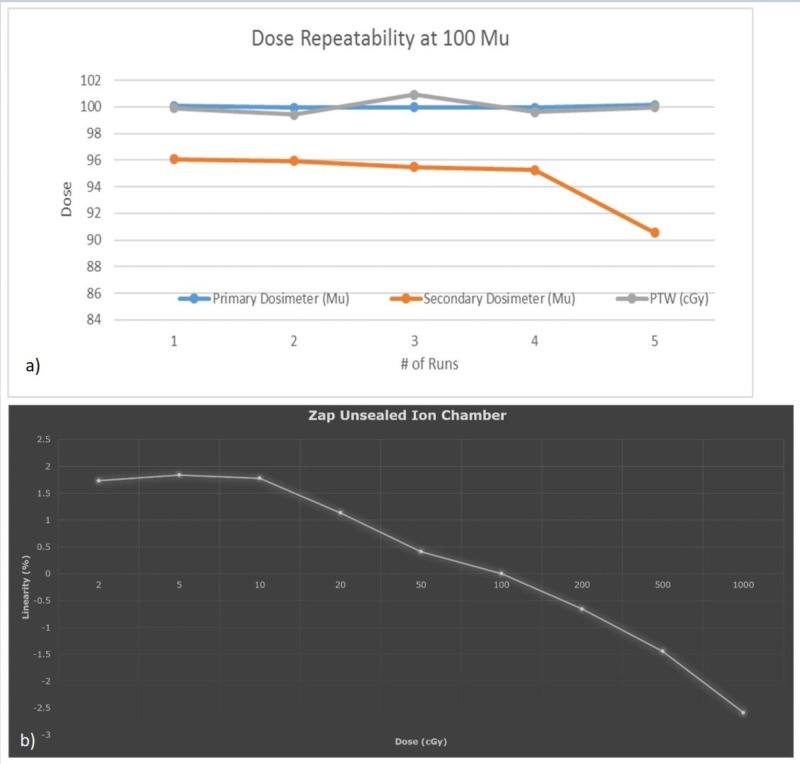
Dose monitor (a) ionization chamber response repeatability (b) and linearity

Dose monitor response reproducibility and linearity were investigated, with an independent PTW absolute dosimetry measurement system (Physikalisch-Technische Werkstaetten, Freiburg, Germany), and the results were found to adhere to IEC 60601-2-1 standards [4]. Figure 13 shows the results of (a) chamber response reproducibility (b) and response linearity, respectively.

It can be concluded that the dose monitor reproducibility remains within +/-2%, while response linearity remains within +/-3% across the range of clinically applicable MUs from 2 MU to 1,000 MU.

Regulations - IEC - and how it relates to system and testing design requirements

The Zap-X system was designed to adhere to all applicable IEC regulations regarding electromagnetic (EM) and mechanical safety, radiation safety, and the functioning and performance of the linear accelerator, as well as the imaging system. IEC standards 60601-2-1, 61217, and 62083 were used to design applicable aspects of the system [4, 7-8]. Affected characteristics are radiation leakage, beam performance, and safety features.

Beam performance specifications and system acceptance testing

The Zap-X Beam performance specifications and system acceptance testing criteria were established based on clinical requirements and system capabilities and were established as follows:

(1) Photon beam energy: Single photon beam energy of 3.0 MV; PDD = 40% ± 2% for 2.5 cm circular field size at 45 cm SSD

(2) Dose rate: 1,500 MU/min at 45 cm source axis distance (SAD); 1 MU = 1 cGy at SAD = 45 cm, 2.5 cm field size, at depth of dose maximum; Linearity shall not exceed ±3 %; Reproducibility of ± 2 MU or 2%, whichever is greater; Orientation dose stability of ± 2 MU or 3%, whichever is greater.

(3) Beam symmetry shall not exceed ± 2%.

(4) The leakage radiation to the patient plane shall be less than 0.1%.

(5) The radiation outside of the patient plane at 1 meter from the beam centerline shall be less than 0.1%.

(6) The collimator transmission shall be less than 1%.

(7) Radiation safety interlocks.

System Acceptance Tests Include the Following Aspects

(1) Mechanical Tests: Axial/oblique gantry Isocentricity: +/- 1.0 mm; Laser position on the physical center of collimator: +/- 1.0 mm; Accuracy of major positions: home/QA, RT/LT lat: +/- 0.5 deg

(2) Dosimetry: Absolute calibration (SAD: 45 cm, d = dmax): 1.0 cGy/MU +/- 2%; Reproducibility (100 MU): +/- 1%; Dose rate stability: 1,500 MU/min +/- 10%; Linearity with dose (2 MU to 1,000 MU): +/- 3%

(3) Beam Performance: accelerating potential by ionization ratio (I20/I10): 0.505 +/-0.05; Energy determination by PDD at SSD = 45 cm: 40.0% +/- 2%; Beam symmetry (25 mm collimator): +/- 2.0%; Field centering around cax: +/- 0.5 mm; Laser coincidence with beam cax: +/- 0.5 mm; Radiation field size equal to stated collimator size: +/- 0.25 mm; Beam penumbra (80% - 20% distance at 10 cm depth): ≤ 4.0 mm

(4) MV Detector: Response reproducibility and linearity shall match dose monitor ionization chamber performance.

(5) kV Imaging: Spatial resolution; Low contrast resolution; Image uniformity; Indication of imaging device center and coincidence with beam cax; Verification of dimensional accuracy (x and y directions).

(6) Safety: Maximum radiation to the patient (compared to cax, at dmax, SAD = 45 cm): ≤ 0.1%; Leakage outside the ZAP housing at 1.0 m distance: ≤ 3.0 mR/hr; Interlock test shell, door, and interrupt buttons.

Beam data and treatment planning system qualification

A complete set of beam data was acquired with the PTW 3D water phantom. The 3 MV photon beam was characterized by acquiring PDD values for all available collimator sizes and to a depth of 20 cm, off-center ratios (OCRs) for all collimator sizes at 0.7 cm (dmax), 2.5 cm, 5.0 cm, 10 cm, 20 cm, and 25 cm, as well as collimator output factors at dmax and SAD = 45.0 cm for all collimator sizes of 4.0, 5.0, 7.5, 10.0, 12.5, 15.0, 20.0, and 25.0 mm.

**Figure 14:**
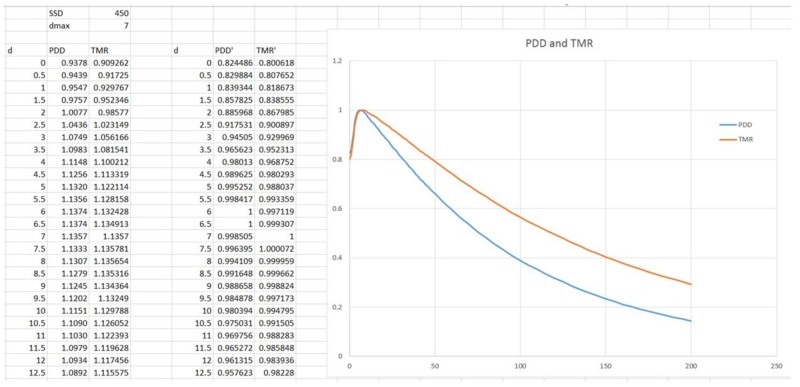
Percent depth dose (PDD) and tissue-maximum ratio (TMR) measurements for the 3 MV Zap-X beam MV: megavoltage

**Figure 15:**
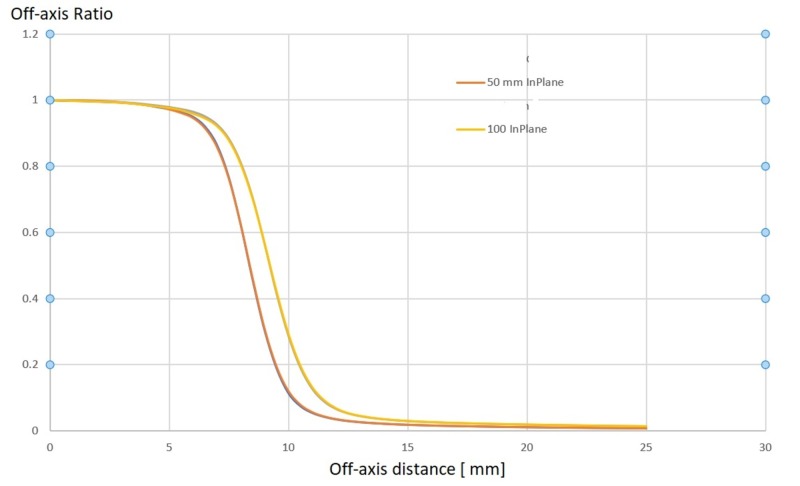
Off-center-ratios for the 3 MV Zap-X beam in-plane and cross-plane at 50 mm and 100 mm depth MV: megavoltage

Sample data sets for the PDD are shown in Figure 14, while OCR values are shown in Figure 15.

**Figure 16:**
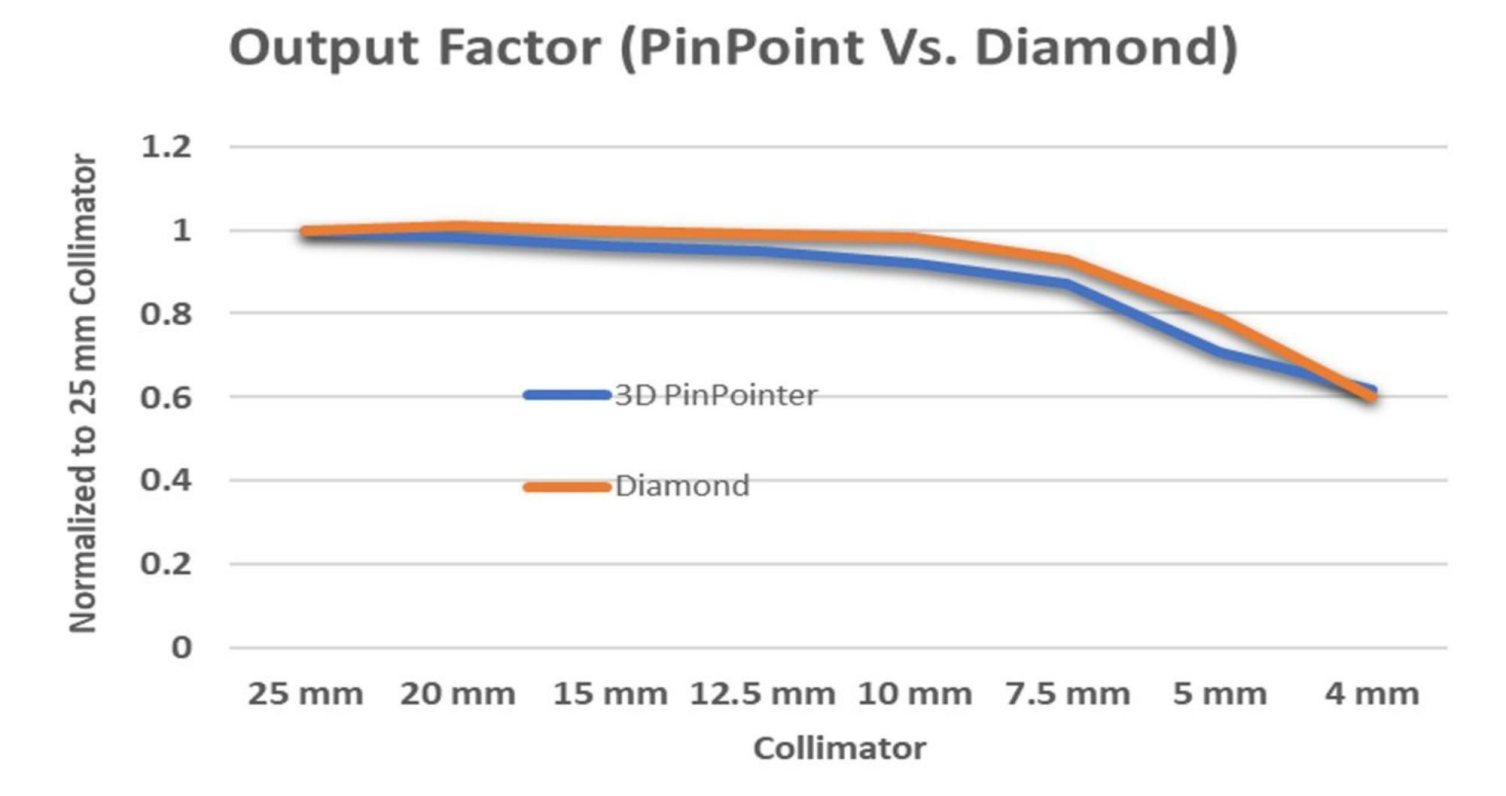
Output factors measured with pinpoint ionization chamber and diamond detector

Figure 16 shows output factors measured with the PTW model 31022 pinpoint ionization chamber and the PTW model TN60019 diamond detector (Physikalisch-Technische Werkstaetten, Freiburg, Germany).

**Figure 17:**
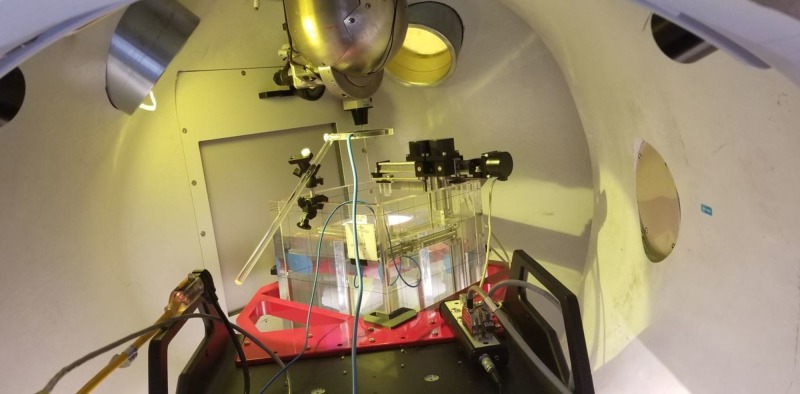
Set-up of 3D PTW water phantom inside the Zap-X treatment capsule 3D: three dimensional

Because the Zap-X has a unique system configuration, we exclusively used a PTW MP3XS 3D water phantom system for beam data measurements. A dedicated supporting frame was used to hold the water phantom, which is mounted securely at the front end of the patient bed with three-level adjustable knobs. The level of the water phantom was adjusted prior to moving into the scanning area. A collimator revolver was configured with eight different collimator holes which are accurately produced beam spot sizes at isocenter ranging from 4 mm, 5 mm, 7.5 mm, 10 mm, 12.5 mm, 15 mm, 20 mm, to 25 mm. The 25 mm collimator is the reference collimator for absolute dose calibration. To level the collimator housing at 25 mm collimator, an alignment kit was used to locate the perpendicular position to the water surface by adjusting the oblique gantry. An isocenter fixture is used to provide the distance required for 45.0 cm SAD and center position for the water phantom. By aligning and adjusting the PTW pointing device with the isocenter fixture using PTW control pendant (Physikalisch-Technische Werkstaetten, Freiburg, Germany), the origin of the scanning system was defined. When the water level was at the tip of the PTW pointer, the pointer was replaced with the PTW dosimetry diode for beam data acquisition (Physikalisch-Technische Werkstaetten, Freiburg, Germany). Because the small beam spot size is used, especially 4 and 5 mm, the smallest PTW ionization chamber 0.016 ccm is needed to get a more accurate measurement for the output factor. For the same reasons, a radiotranslucent PTW transmission reference chamber (Physikalisch-Technische Werkstaetten, Freiburg, Germany) is used. The 3D PTW water phantom is shown in Figure 17.

**Figure 18:**
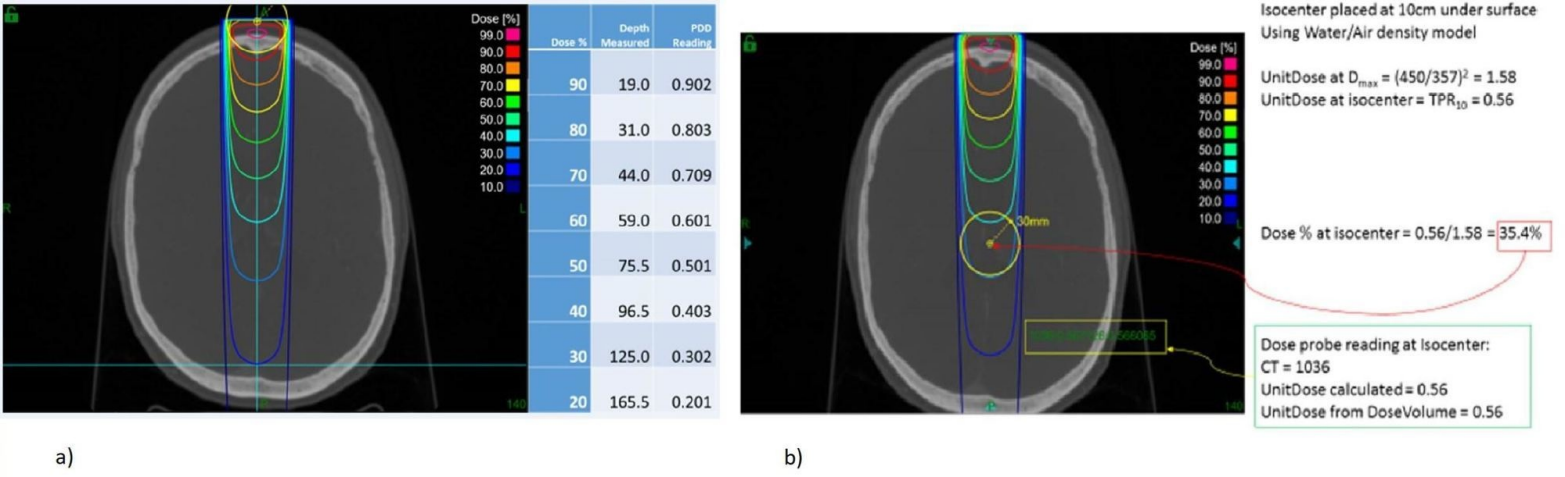
Verification of a) PDD and b) TMR values on the cax PDD: percentage depth dose; TMR: tissue-maximum ratio

**Figure 19:**
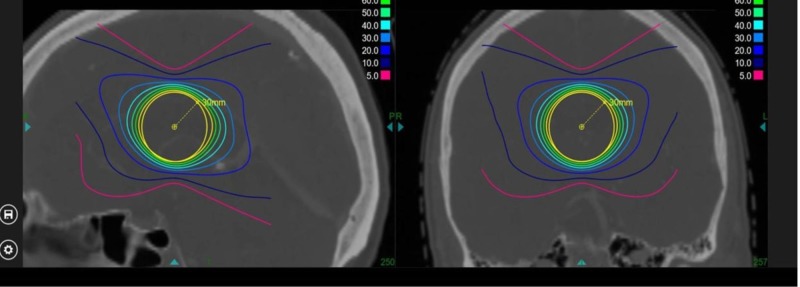
Verification of treatment plan with full beam set

The functionality and accuracy of the treatment planning system were verified by independently calculating expected dose in various scenarios: single beam SSD setup (Figure 18A), single beam source axis distance (SAD) setup (Figure 18B), and two beam set, isocentric setup (Figure 19). The calculated expected dose was computed using the following formula:

D(x) = 1 (cGy/MU) x OCR (coll, deff, OAD) x TPR (coll, deff) x OF (coll) x (450/SAD)2,

where:

 x = position where the dose is calculated;

 coll = collimator size;

 deff = effective depth along the central axis for position x;

 OAD = off-axis distance from x to the axis;

 SAD = distance from the source to the projection point of x on the central axis.

The single isocenter conformality indices for the prescription isodose line, the 80%, 70%, 60%, 50%, 40%, and 30% isodose lines, were calculated in relation to the target volume and are shown in Table 3.

**Table 3:** Conformality indices for prescription isodose line, 80%, 70%, 60%, 50%, 40%, and 30% isodoses Normalized to the 80% isodose volume. cm^3^: cubic centimeter

Prescription Iodose Line [%]	Volume covered (cm^3^)	Conformality index
80	13.38	1.0
70	16.51	1.23
60	19.95	1.49
50	24.57	1.84
40	32.30	2.41
30	48.73	3.64

The calculated expected dose was compared with the dose predicted by the treatment planning system and found to be acceptable according to the American Association of Physicists in Medicine (AAPM) guidelines.

Quality assurance program

A complete set of principal QA procedures was developed and implemented, including daily output verification, weekly beam performance verification, monthly output calibration, and an annual calibration for verification of baseline data established during the commissioning of the system.

Daily

Door interlock and key switch interlocks; ‘Rad-On’ light tests; Collimator selection test; Warm-up 6,000 MU; Verify absolute calibration of linear accelerator dose for largest collimator size; Test laser alignment with isocenter pointer; Test full functionality of the system, pre-programmed: all rotations; Report all functionally relevant operating parameters: vacuum, dose rate, magnetron performance, and other linear accelerator operating parameters; Daily Winston-Lutz type positioning test

Weekly

Linear accelerator field symmetry; End-to-end (E2E) test for the largest collimator

Monthly

Calibration verification of absolute dose with ADCL certified ionization chamber; Ionization ratio verification with a dedicated solid water phantom

Quarterly

Imaging system alignment test; Image system quality tests; Patient table positioning tests according to the AAPM Task Group (TG) 101 [9]

Annually

Full calibration of absolute dose according to the AAPM TG 51 [10]; PDD, collimator output factor, off-center ratio measurements; Verification of data used in the treatment planning system (TPS); Verification of TPS functionality and calculation accuracy; Complete E2E tests, including the humanoid RANDO® Phantom (Imaging Solutions, Pty Ltd., Shailer Park, Australia): computed tomography (CT) imaging, transfer to TPS, treatment planning, patient-specific QA, delivery of plan, evaluation of accuracy of delivered dose distribution, and absolute dose measurement with the ionization chamber embedded in the phantom. Complete safety systems evaluation, positioning of the collimator revolving wheel, collision avoidance patient-to-collimator, and couch positioning restrictions.

**Figure 20:**
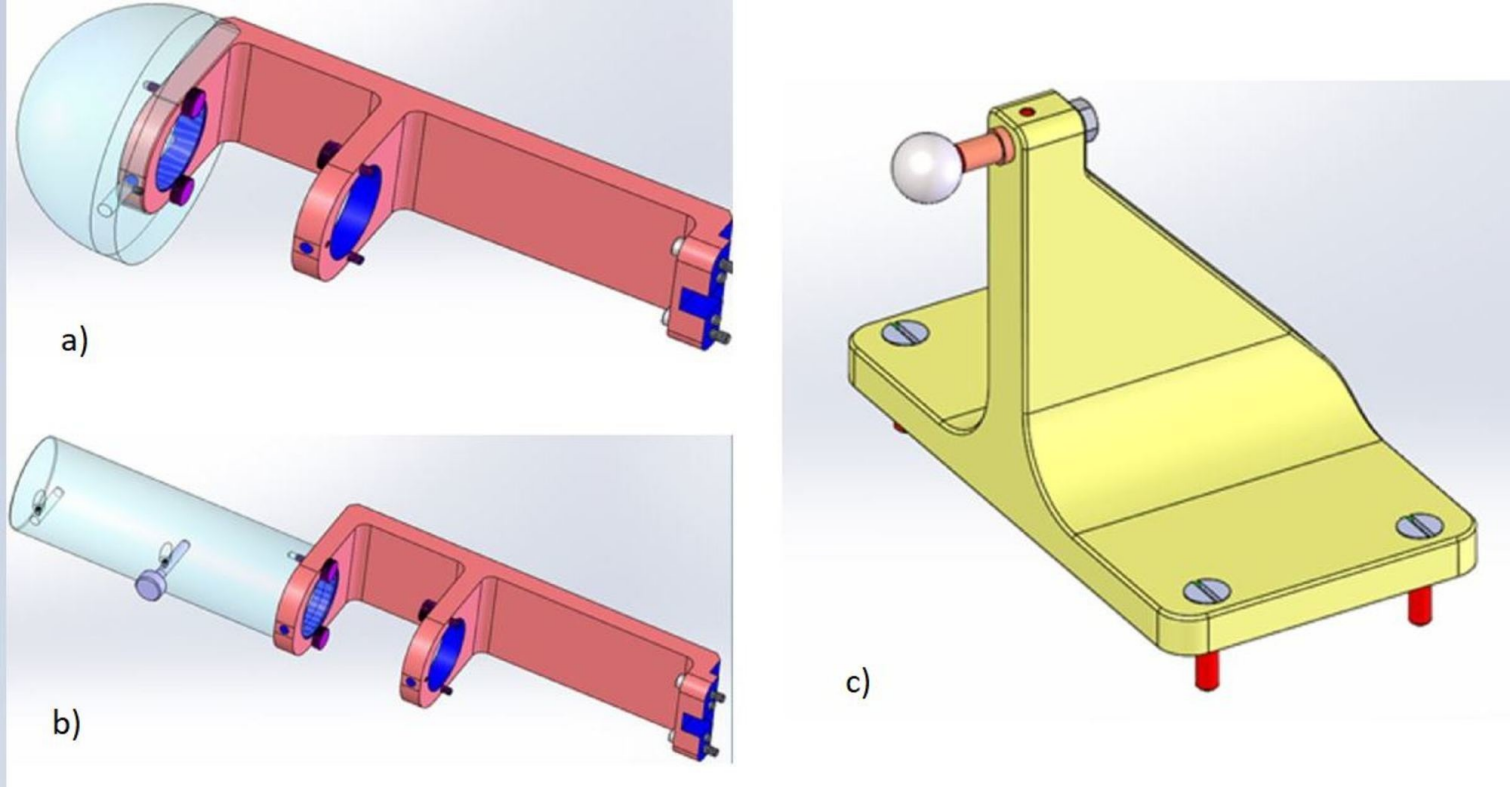
Zap-X quality assurance tools used for a) absolute dose calibration, b) ionization ratio measurement, and c) radiation isocenter verification

Figure 20 below shows Zap-X-specific QA tools.

**Figure 21:**
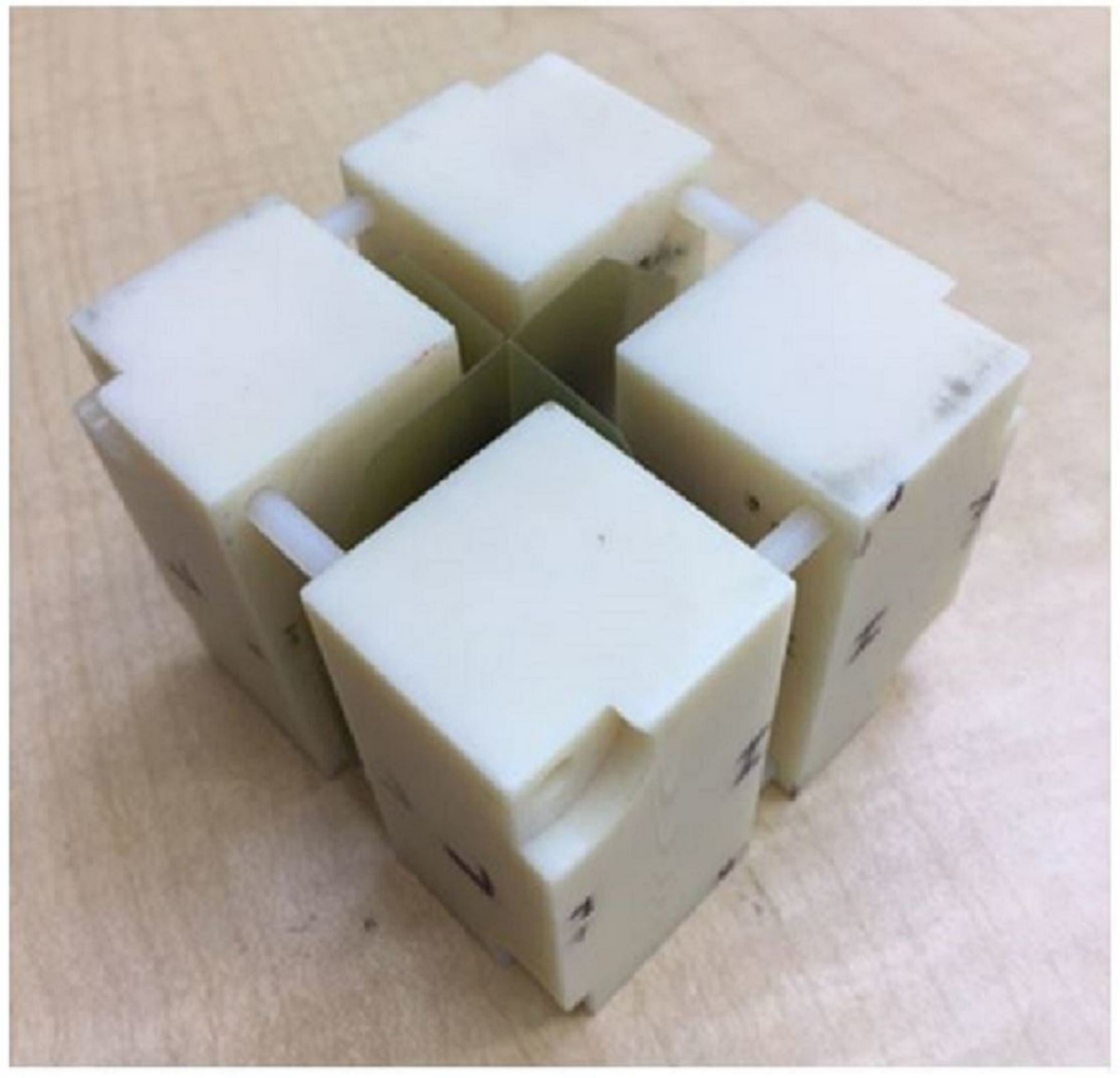
Gafchromic film embedded in the ball cube for use in the phantom

**Figure 22:**
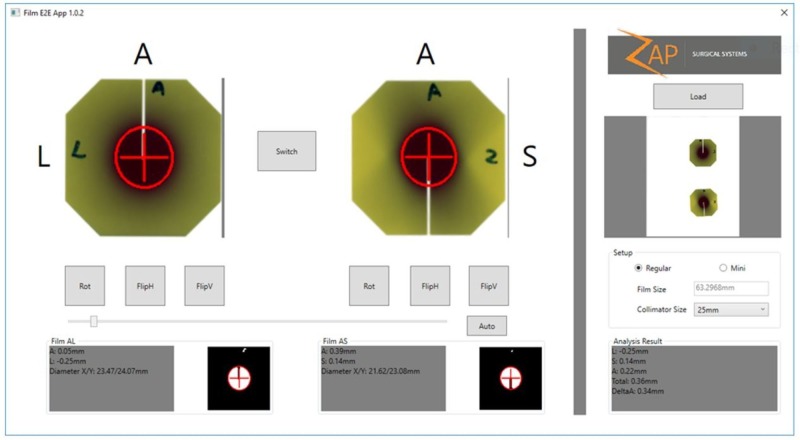
Results of the end-to-end (E2E) test

To verify the overall system accuracy, a weekly end-to-end test will be performed that includes CT imaging, contouring, treatment planning, treatment delivery, and treatment evaluation. The Gafchromic film is inserted into the ball cube that is embedded inside the anthropomorphic skull phantom (Figure 21). After the treatment dose exposure, the film will be evaluated to determine the center of the exposure sphere in relation to the center of the ball cube. The results are shown in Figure 22.

## Discussion

As no standards exist for external beam-based dedicated intracranial SRS systems at this treatment energy, the above-presented results will serve as a set of baseline parameters for the new class of specialized radiation treatment devices. Additional parameters, such as reproducibility of parameters for subsequent production systems and individual long-term stability, remain to be explored and determined.

## Conclusions

The Zap-X system was found to meet safety, accuracy, and performance requirements widely accepted in the radiation oncology and radiosurgery industry. Furthermore, the system has been shown to meet the practical clinical needs of the radiosurgery community.
